# Women’s right to science and health: 30 years on, progress and gaps remain

**DOI:** 10.1093/inthealth/ihag011

**Published:** 2026-03-10

**Authors:** Sandrine Hakiza, Silvia Ferazzi

**Affiliations:** Advocacy Department, Medicines for Malaria Venture, 1215 Geneva, Switzerland; Advocacy Department, Medicines for Malaria Venture, 1215 Geneva, Switzerland

## Background

The Beijing Declaration and Platform for Action (BPfA)^1^, adopted by 189 governments at the Fourth World Conference on Women in September 1995, remains the most comprehensive global blueprint for advancing women’s rights and achieving gender equality. Building on and aiming to accelerate the implementation of the 1985 Nairobi Forward-Looking Strategies, established during the World Conference to Review and Appraise the Achievements of the United Nations Decade for Women, the BPfA recognized that the human rights of women and girls are an inseparable part of universal human rights.^1^

Among its 12 critical areas of concern, the BPfA identified women and health as a central priority. It noted that medical research and epidemiological studies have often been gender-biased, based solely on the realities of men, and highlighted the importance of advancing women’s equal access to science, particularly through their inclusion in health research and product development, to address sex and gender-specific health needs. Advancing women’s participation in science, especially in health research and product development, was framed not as a technical necessity, but as a matter of justice.^1^

To that end, the BPfA’s strategic objective C.4, *Promote research and disseminate information on women’s health*, proposed concrete measures to be taken to advance women’s access to science and health, including^2^:

1. train researchers and introduce systems that allow for the use in policy-making of data collected, analysed and disaggregated by, among other factors, sex and age, other established demographic criteria and socio-economic variables;
2. promote gender-sensitive and women-centred health research, treatment and technology and link traditional and indigenous knowledge with modern medicine, making information available to women to enable them to make informed and responsible decisions;
3. increase the number of women in leadership positions in the health professions, including researchers and scientists, to achieve equality.

## Thirty years of progress, and persistent barriers

Implementation of the various objectives of the BPfA is evaluated by the Commission on the Status of Women (CSW) every five years, with each review resulting in an outcome document in which countries establish their commitment to continue their efforts.^3^

Progress since 1995 has been substantial. Laws against gender-based violence have expanded from just 12 countries to 193, with some countries updating their laws to include new forms of violence linked to technology.^4^ Educational gender gaps have narrowed dramatically, and most nations now ban workplace discrimination. Furthermore, national and regional policy changes, such as regulatory guidance on the inclusion of pregnant and breastfeeding women in clinical research,^1^ have helped increase attention to sex and gender differences in health research and improve women’s representation in research teams and leadership. However, challenges persist, such as continued gender bias in health research, which lead to gaps in women’s access to information and data, which impact on the availability of medicines adapted to their needs. Furthermore, women continue to be under-represented in leadership roles, and funding for their inclusion in science and research remains limited. Unfortunately, these developments have not been systematically tracked within formal BPfA implementation reviews, underscoring ongoing gaps in accountability for strategic objective c.4 and for women’s right to science more broadly.

## A spotlight on accountability: a CSW69 side event

Marking 30 years since the BPfA’s adoption, a side event at the sixty-ninth session of the Commission on the Status of Women (CSW69), co-hosted by Medicines for Malaria Venture (MMV), Speak Up Africa, and Treatment Action Group (TAG), brought these issues to the forefront. The event convened global health and human rights experts to assess progress and gaps in women’s right to science and health, and push for renewed action.

The meeting aimed to:

1. review progress on BPfA commitments, particularly women’s inclusion in science and health innovation;
2. strengthen accountability for implementing the BPfA and CSW69 Political Declaration;
3. mobilize civil society and other actors to advocate for gender-responsive health research and policy.

The discussion offered a stark reminder of what is at stake. ‘Whether it’s explicit or incidental, the exclusion of women from clinical trials results in real health disparities and harms’,^5^ emphasized Mike Frick of TAG. For decades, pregnant women had been systematically barred from tuberculosis research. As a result, lifesaving evidence was delayed: it was only in 2019 that a common preventive therapy, isoniazid preventive therapy, was found to increase adverse pregnancy outcomes,^6,7^ a finding that could have surfaced 60 years earlier. Similar patterns persist in HIV cure trials, where women make up less than 20% of participants^8^ despite bearing a higher global burden of the disease.

## Inclusion by design: emerging models

Examples from Liberia and the malaria field demonstrate how inclusion can work in practice. In Liberia, post-war reforms, driven in part by CSW advocacy, established gender-focused task forces that later helped expand malaria prevention in pregnancy. Local, community-led initiatives raised uptake of intermittent preventive treatment in pregnancy (IPTp) from 4% in 2023 to 11.4% in 2024.^9^

Meanwhile, Andrea Lucard presented a compelling case of how MMV’s MiMBa (Malaria in Mothers and Babies) initiative embodies inclusion by design by embedding sex and gender considerations from early drug discovery to regulatory approval. MiMBa has reshaped the approach to women’s participation in research, including MMV's groundbreaking SAFIRE trial, the first interventional study of antimalarials in first-trimester pregnancy. This influence is also evident in concrete regulatory commitments: eleven African regulatory authorities convened in Rwanda in 2025 to discuss harmonized standards for including pregnant and breastfeeding women in clinical trials and pledging to form a working group to coordinate comments on the forthcoming 2025 ICH E21 guidelines.^10^

## From obstacles to opportunities

Yet these advances remain fragile. Patricia Kamara of the Christian Health Association of Liberia reminded participants that when donor funding was abruptly withdrawn, community health efforts nearly collapsed, forcing reliance on local taxation and patchwork solutions. Without sustainable financing and national political will, gender-responsive health systems remain vulnerable.

Barriers extend beyond budgets. Speakers noted how regulatory language often perpetuates bias, from trial designs built around male biomarkers to ethics boards that still define pregnant women as ‘vulnerable subjects’^11^ rather than rights holders. Such frameworks continue to marginalize the very populations most affected by disease.

The Ebola outbreak offered a painful illustration: during the 2014–2016 West African epidemic, pregnant women were denied access to experimental therapies and vaccines deemed ‘too risky’, despite previous Ebola outbreak data showing 89% maternal mortality and 100% foetal/neonatal mortality, as well as ethics committee requests for their inclusion on the grounds of justice and equity.^12^ The risk, in truth, lay in their exclusion. This tragedy catalysed change and during the 2018–2020 DRC Ebola outbreak, advocacy efforts led the DRC National Ethics Committee and the WHO Strategic Advisory Group of Experts on Immunization to reverse course in February 2019, authorizing vaccination of pregnant and lactating women. However, this delay in reversal likely cost lives and demonstrates that advocacy can change deadly policies, but such changes come too late when they happen mid-crisis rather than being embedded in trial design from the start.

## A roadmap for tangible action

The discussion closed with a call-to-action centring on three pathways:

1. **Mainstream the right to science** in policy and advocacy, by positioning it alongside other core human rights. Participants emphasized cross-disease learning, such as adopting HIV inclusion frameworks for tuberculosis and malaria research, to overcome structural barriers.
2. **Advocate at multiple levels**, linking global policy platforms like Beijing+30 with targeted engagement of regulators, insurers and ethics boards.
3. **Invest in political will and local leadership**, urging governments to fund science, technology, engineering and mathematics (STEM) opportunities for women and scale community health models, like Liberia’s IPTp initiative, through South–South collaboration.

## From Beijing to 2030: a collective responsibility

As the Beijing+30 review process unfolds, the message is unequivocal: women’s right to science is inseparable from their right to health, and realizing this vision will require collective action. Excluding women, particularly pregnant and breastfeeding women, from research is not just a missed opportunity for better medicine, it is a violation of their basic human right to enjoy the advancements of science.

Sustained advocacy, stronger partnerships and renewed political will can bring the global community closer to the BPfA’s vision of equality, where every woman and girl benefits fully from scientific progress by 2030.

## Data Availability

No new data were generated or analysed in support of this article.

## Footnotes

1 Examples of progressive regulatory policy changes include Article 33 of the 2014 EU Clinical Trials Regulation, the 2018 FDA guidance on including pregnant women in clinical trials and the 2025 ICH E21 guideline on the inclusion of pregnant and breastfeeding individuals in clinical research.
